# Correction to “Persistent Sheaf Laplacian Analysis of Protein Flexibility”

**DOI:** 10.1021/acs.jpcb.5c03679

**Published:** 2025-06-06

**Authors:** Nicole Hayes, Xiaoqi Wei, Hongsong Feng, Ekaterina Merkurjev, Guo-Wei Wei

An error was discovered in the computation of average Pearson correlation coefficient (PCC) values for our PSL model on the data sets in Table 1 of our manuscript. The PCC values for individual proteins were not affected, but the original average computation mistakenly excluded a portion of the proteins in each data set due to a data formatting error. We have corrected the formatting issue so that all PCC values are included in computing the average PCC for each data set. A corrected version of Table 1 is below, with updated values bolded.

**Table 1:** Average Pearson Correlation Coefficients for the PSL Model Compared to Other Methods^a^

Protein Set	PSL	ASPH (B)^5^	ASPH (W)^5^	opFRI^25^	pfFRI^25^	GNM^14^	NMA^14^
Small	**0.854**	0.85	0.86	0.667	0.594	0.541	0.480
Medium	**0.710**	0.69	0.69	0.664	0.605	0.550	0.482
Large	**0.649**	0.61	0.62	0.636	0.591	0.529	0.494
Superset	**0.682**	0.65	0.66	0.673	0.626	0.565	NA

a Experiments were conducted on the full set of 364 proteins as well as three subsets of small, medium, and large protein structures as described by Park et al.^14^ ASPH denotes the atom-specific persistent homology method developed by Bramer et al.,^5^ with results using Bottleneck (B) and Wasserstein (W) metrics displayed. Both sets of ASPH results used both an exponential and Lorenz kernel for least-squares fitting. opFRI and pfFRI results are from Opron et al.,^25^ and GNM and NMA results are from Park et al.^14^

Given the change in mean PCC value for the superset, the authors also amend the comparison to GNM in the abstract, which should now read: “Our PSL model demonstrates an increase in accuracy of 20% compared to the classical Gaussian network model (GNM) in predicting *B*-factors for a data set of 364 proteins.”

Additionally, the following sentences appear at the end of Section 2.1.2 of the original manuscript: “The PSL model achieves improved performance over all other compared methods on all data sets shown in Table 1. In particular, the PSL model improves the benchmark GNM by 32%.” We correct this portion of the discussion to “The PSL model achieves improved performance over most other compared methods on the data sets shown in Table 1. In particular, the PSL model improves the benchmark GNM by 20%.”

All other results in the original manuscript are unchanged, including those in the individual protein case studies and the blind prediction experiments. Furthermore, apart from the change in percent improvement compared to GNM, all general conclusions presented in our paper remain the same.

